# Effects of Chronic and Acute Intranasal Oxytocin Treatments on Temporary Social Separation in Adult Titi Monkeys (*Plecturocebus cupreus)*

**DOI:** 10.3389/fnbeh.2022.877631

**Published:** 2022-06-22

**Authors:** Rocío Arias del Razo, Maria de Lourdes Velasco Vazquez, Petru Turcanu, Mathieu Legrand, Allison R. Lau, Tamara A. R. Weinstein, Leana R. Goetze, Karen L. Bales

**Affiliations:** ^1^Department of Psychology, University of California, Davis, Davis, CA, United States; ^2^California National Primate Research Center, Davis, CA, United States; ^3^Department of Statistics and Informatics, Universidad Veracruzana, Xalapa, Mexico; ^4^CNRS, LNCA UMR 7364, Strasbourg, France; ^5^Centre de Primatologie de l’Université de Strasbourg, Niederhausbergen, France; ^6^Animal Behavior Graduate Group, University of California, Davis, Davis, CA, United States; ^7^Department of Neurobiology, Physiology, and Behavior, University of California, Davis, Davis, CA, United States

**Keywords:** intranasal oxytocin, pair bonds, separation distress, vocalizations, contact

## Abstract

In socially monogamous titi monkeys, involuntary separation from a pair mate can produce behavioral distress and increased cortisol production. The neuropeptide oxytocin (OXT) is thought to play an important role in the separation response of pair-bonded species. Previous studies from our lab have shown that chronic intranasal oxytocin (IN OXT) during development can have long-term effects on adult social behavior. In the current study, we examined the chronic and acute effects of IN OXT or Saline (SAL) on the subjects’ response to a brief separation from their pair mates. Subjects with a history of chronic IN OXT or SAL treatment during development received a single dose of OXT or SAL as adults 30 min before being separated from their pair mate. Chronic treatment consisted of a daily dose of IN OXT (0.8 IU/kg) or SAL (control) from 12 to 18 months of age. Subjects (*N* = 29) were introduced to a pair mate at 30 months of age. After the pairs had cohabitated for 5 months, pairs underwent two “Brief Separation” (OXT and SAL) and two “Non-Separation” (OXT and SAL) test sessions. Vocalizations and locomotion were measured as behavioral indices of agitation or distress during the Brief Separation and Non-Separation periods (30 min each). We collected blood samples after the Brief Separation and Non-Separation periods to measure cortisol levels. Our results showed subjects treated with chronic OXT had a reduction in long call and peep vocalizations compared to subjects treated with chronic SAL. Subjects treated with chronic SAL and acute OXT produced more peeps and long calls compared to animals treated with acute SAL; however, patterns in this response depended on sex. Cortisol and locomotion were significantly higher during the Brief Separation period compared to the Non-Separation period; however, we did not find any treatment or sex effects. We conclude that chronic IN OXT given during development blunts the separation response, while acute OXT in chronic SAL subjects had sexually dimorphic effects, which could reflect increased partner seeking behaviors in males and increased anxiety in females.

## Introduction

Pair bonds in monogamous species are formed and maintained by an array of different biopsychological processes, including attraction, reward, aversion towards outsiders, and distress upon separation (Bales et al., 2021; Beery et al., 2021). Separation distress is a defining characteristic of an attachment bond (Bowlby, 1973; Hazan and Shaver, 1987) and helps to maintain the bond. The neurobiological response to the separation of a monogamous pair has been best studied in prairie voles (*Microtus ochrogaster*, Bosch et al., 2009; Pohl et al., 2019). Separation from the female pair mate in male prairie voles leads to a suite of behaviors referred to as “passive stress-coping”, which are indicative of depressive-like behavior (e.g., floating instead of swimming in a forced swim test). Exogenous administration of OXT reduces the stress-coping behaviors, while an OXT antagonist or silencing of OXT receptor signaling can increase these behaviors even in the presence of the partner (Bosch et al., 2016).

Our animal model the titi monkey, is a socially monogamous and biparental species. Adults can establish long-lasting pair bonds characterized by a strong preference for their familiar partner (Carp et al., 2016; Rothwell et al., 2020), mate-guarding behavior (Fisher-Phelps et al., 2016; Witczak et al., 2018), territorial defense against intrusion (Mason, 1966), distress upon separation (Mendoza and Mason, 1986) and the ability of the partner to buffer against stress (Mendoza et al., 2000). The pair bond is considered to have some degree of strength or quality when it is specific to a particular individual, both individuals are sexually mature, and the pair-bonding behaviors mentioned above are displayed in association with the chosen partner, rather than indiscriminately among conspecifics (Bales et al., 2021).

In titi monkeys, an acute involuntary separation from the pair mate triggers an increase in vocalization, locomotion, and cortisol (Mendoza and Mason, 1986). Neurobiological changes are dependent upon the duration of the separation (48 h or 2 weeks) but include a sustained increase of OXT in the cerebrospinal fluid, plasma cortisol, plasma insulin, and a reduction of cerebral glucose uptake in areas involved in the OXT and vasopressin (AVP) systems (Hinde et al., 2016). Continued or permanent separation from the pair mate is known to cause significant distress (Mendoza et al., 2000). Titi monkeys can show sustained glucocorticoid elevations in response to a long-term separation (at least 30 days) from attachment figures. This response decreases when the monkeys are returned to their respective attachment figures or upon the formation of a new heterosexual pair (Mendoza et al., 2000).

Effects of long-term separations from pair mates have also been studied in socially monogamous rodents and humans. In male prairie voles, long-term separation from female partners elicits anxiety-like and depression-like behaviors, disrupts bond-related behaviors, and alters neuropeptide systems that regulate such behaviors (Sun et al., 2014). In humans, the subsequent grief may have substantial impacts on health and survivorship (Kaprio et al., 1987; Chen et al., 1999; Szuhany et al., 2021). In contrast, the presence of a close social partner during or subsequent to a stressor provides important health benefits by attenuating the magnitude of the HPA-axis response, and boosting a faster recovery, a phenomenon known as social buffering (Smith et al., 1998; Kikusui et al., 2006; DeVries et al., 2007).

The OXT system has been identified to have a leading role in the neuromechanisms associated with social buffering (Smith and Wang, 2012) and in reducing stress-induced activation of the HPA axis (Neumann et al., 2000). Genetic variation of the OXT receptor gene (OXTR) has also been shown to modulate the effectiveness of positive social interaction as a protective buffer against stressful experiences (Chen et al., 2011). Other studies have also found the OXT system to be indirectly involved in alleviating the stress response by inducing partner or social seeking behavior (Cavanaugh et al., 2016; Sicorello et al., 2020) and receiving social support (Heinrichs et al., 2003). Anxiolytic effects of OXT occur after both endogenous release and exogenous OXT administration (Neumann, 2008). In prairie voles, microinjections of OXT in the paraventricular nucleus (PVN) of the hypothalamus decreased anxiety-like behaviors and reduced circulating levels of corticosterone in female voles recovering alone from an immobilization stress task; while injections of an OXT receptor antagonist increased anxiety-like behaviors and elevated circulating levels of corticosterone in female voles recovering with a male partner (Smith and Wang, 2014).

IN OXT is a convenient and non-invasive method of delivering exogenous OXT to specific brain regions where oxytocin acts to impact behaviors (Lee et al., 2020). IN OXT has been shown to have anxiolytic properties in different model species. In adult female squirrel monkeys, repeated administration of IN OXT prior to acute social isolation attenuated the adrenocorticotropic hormone (ACTH) response to stress (Parker et al., 2005). In marmosets, administration of IN OXT did not alter cortisol or behavior in response to a stressor; however, marmosets treated with OXT antagonists had higher HPA-axis activity than when treated with saline (Cavanaugh et al., 2016). In humans, IN OXT increased calmness and decreased anxiety scores during a stress procedure compared to placebo (Heinrichs et al., 2003). Moreover, participants who received social support and IN OXT exhibited the lowest cortisol concentrations during stress exposure, whereas subjects who received no social support and placebo demonstrated the highest cortisol response (Heinrichs et al., 2003).

Understanding the long-term effects of chronic administration is key to considering IN OXT as an anxiolytic treatment option for humans. Studies using repeated administration of IN OXT over an extended period are still scarce and those carried out have indicated long-lasting effects of treatment (Bales et al., 2013; Guoynes et al., 2018; Arias del Razo et al., 2020, 2022; Bernaerts et al., 2020). Additionally, chronic IN OXT effects can differ from single-dose effects, differ between female-male subjects, and dosage (Bales et al., 2013; Huang et al., 2014; Arias del Razo et al., 2020, 2022). Effects of IN OXT can also be predicted by pretreatment of OXT concentrations in blood (Parker et al., 2017). Furthermore, long-term effects in behavior have been reported up to one month and even one-year post-treatment (Arias del Razo et al., 2020, 2022; Bernaerts et al., 2020) and compensatory changes in the OXT and AVP systems (Guoynes et al., 2018).

This research is a continuation of previously published studies on the behavioral and neural effects of chronic IN OXT in juvenile animals (Arias del Razo et al., 2020) and on the subsequent long-term effects in adult social behavior (Arias del Razo et al., 2022). To provide some background on the subjects of this study, we summarized here the main findings from both studies. During chronic intranasal OXT treatment, male juveniles treated with chronic OXT displayed an increased preference to spend time in proximity to a strange adult pair, without reducing the time they spent in proximity to their parents; however, males also showed an increase in non-social anxiety-like behavior. Immediately following OXT administration, both male and female juveniles exhibited higher glucose uptake in areas that have been shown to be involved in primate selective social behavior (paternal and pair mate attachment) and/or express OXTR and AVPR1a: anterior cingulate cortex (ACC), lateral septum (LS), caudate nucleus (C), nucleus accumbens (NACC), putamen (PUT), amygdala (AMY), hippocampus (HIPP), paraventricular nucleus of the hypothalamus (PVN), and supraoptic nucleus of the hypothalamus (SON; Arias del Razo et al., 2020). OXT-treated females showed earlier ovarian activity, and OXT-treated males showed a transient increase in androgens during development (Conley et al., 2022). As paired adults, 1-year post-treatment, OXT-treated animals—particularly males—showed higher affiliative behavior towards their pair-mate and boldness in multiple contexts. Chronic IN SAL animals and chronic IN OXT treated males successfully formed a preference at 1-week post-pairing by showing a significant preference for their partner during a partner preference test validated for titi monkeys (Carp et al., 2016; Rothwell et al., 2020). However, chronic IN OXT females did not form a preference at 1-week post-pairing; it was not until 4 months post-pairing, that both chronic OXT and chronic SAL females and males had successfully formed a preference for their partner. IN OXT-treated males also showed a larger effect size in time spent touching their partner’s grate during the test (a measure of the attempt to establish physical contact; Arias del Razo et al., 2022).

For this study, we considered three main types of titi monkey vocalizations: peeps, long calls, and duets. Peeps are short and repetitive pulse-like vocalizations that usually occur in a succession of multiple pulse notes. Peeps are sometimes referred to as “isolation peeps” or “alarm calls” typically heard when a subject is in distress or separated from their figure of attachment (parent/partner). Long calls are the male and female contributions to a duet; a duet is typically coordinated between partners or older juveniles and parents. Long calls are wideband vocalizations with a prominent low-frequency component. Long calls and duetting are used to define and reinforce territorial boundaries and decrease the need to participate in costly territorial behaviors, as well as bonding and mate guarding (Marshall and Marshall, 1976; Robinson, 1981; Marshall-Ball et al., 2006). A recent study by Lau et al. (2020) showed that adult titi monkeys have individually distinct patterns of morning duet vocalizations, suggesting that animals likely benefit from individual recognition. The ability to identify conspecifics aurally may be important in the wild, where titi monkeys often cannot see or smell each other from long distances and must rely on acoustic signaling for conspecific recognition (Robinson, 1981; Lau et al., 2020). Other common adult vocalizations include peep introductions and long call introductions. Peep introductions appear immediately before a set of peeps, they are defined as single phrases centered at 5.5 kHz ± 1.5 kHz, have characteristic frequency modulation, an upward sweep, and are longer (>250 ms) than the duration of individual peeps. Long call introductions appear either alone or immediately before a long call, often showing a frequency modulation comparable to a peep introduction but differ from the latter by a less prominent or altogether lacking upward sweep. The functionality of peep introductions and long call introductions is less defined.

The goal of our study was to investigate the chronic and acute effects of IN OXT on separation distress and affiliation upon reunion in adult pair-bonded titi monkeys. Our subjects were previously treated with chronic IN OXT or SAL as juveniles. We examined the subjects’ response to a brief separation from their pair mate 30 min after receiving a single dose of IN OXT or SAL. We expected subjects that received chronic IN SAL and acute IN OXT to experience a reduced stress response to the brief separation compared to other groups, specifically fewer vocalizations, locomotion, and lower levels of cortisol. For subjects that received chronic IN OXT and acute IN OXT, we expected the acute IN OXT to have a dampened anxiolytic effect, with subjects having a moderate stress response, displaying more vocalizations, locomotion, and higher levels of cortisol as it is possible that the prolonged over-stimulation of the chronic OXT treatment to cause downregulation of receptors (Gimpl and Fahrenholz, 2001; Conti et al., 2009). We also expected OXT treated males to have stronger effects on behavior and cortisol, as males appear more responsive to OXT and females show more resilience to developmental manipulations in the OXT system (Bales and Carter, 2003; Carter, 2003; Bales et al., 2013; Arias del Razo et al., 2020, 2022).

## Methods

### Housing and Subjects

For this study, we used 29 coppery titi monkeys (*Plecturocebus cupreus*, previously *Callicebus cupreus*), OXT = eight females, seven males; SAL = seven females, seven males. All the animals used in this study were born and raised at the California National Primate Center (CNPRC) in Davis, California. Animals were housed in indoor home cages measuring 1.2 m × 1.2 m × 2.1 m or 1.2 m × 1.2 m × 1.8 m cages. Rooms were maintained at 21°C on a 12:12 light-dark cycle (lights on at 6:00 a.m. and off at 6:00 p.m.). Animals were fed twice daily at 8:00 a.m. and 1:30 p.m., and additional enrichment was provided twice a day. For further details of husbandry practices, see Tardif et al. (2006). The University of California Davis Institutional Animal Care and Use Committee approved all housing conditions and experimental procedures described in this article.

We included in the sample all animals that were born in the colony during the time of the study. Within sex we alternated chronic treatment group assignments; the only exception was when two juveniles were from the same family, in which case we assigned them to different treatment groups. We used several color codes throughout the study to represent OXT and SAL, with the purpose of creating additional blinding and avoiding bias. Dosing, data collection, scoring, and analysis were done without the knowledge of the treatment.

While subjects received the chronic treatment, we examined juveniles’ interactions with family members in the home cage, we tested for social preference for parents vs. strangers (Parent Preference Test), anxiety-like behavior (Novel Pattern Test), and changes in brain glucose uptake using ^18^FDG positron emission tomography (PET) scans at 13 months of age. For further details on dosing, behavioral tests, and imagining studies performed while subjects received treatment and results, see Arias del Razo et al. (2020).

From birth to 30 months of age, subjects were housed with both parents and siblings (if present). At 30 months of age (895 ± 12 days, mean ± standard error), subjects were removed from their family group, moved into a new home cage, and paired with an unfamiliar partner of the opposite sex with previously proven fertility (subjects were reproductively naïve). Subjects were removed from their natal group the same day they were paired. New pairs were monitored closely for the first two days to ensure compatibility. If displays of low aggression (chase, grab) and/or distress were observed between the pairs, the initial monitoring period was extended as necessary. In three cases (two SAL-treated females and one OXT-treated male), pairs were separated on the first day of pairing and moved into two adjacent cages connected by a grated window (grate pairing) to allow visual and olfactory access with limited physical contact for 1 week before attempting a second full pairing. In all cases, the aggression was elicited by the males in the pair. These second pairing attempts were successful in all three cases. For further details on the pairing process see Arias del Razo et al. (2022).

To assess the long-term effects of IN OXT on pair-bonding behavior, we examined pair affiliative behavior in the home cage during the first four months and tested for behavioral components of pair-bonding at 1 week and 4 months post-pairing (Partner Preference Tests and Mirror Test). We also assessed long-term changes in brain glucose uptake using ^18^FDG positron emission tomography (PET) scans at 23 (pre-pairing) and 33 (post-pairing) months of age. For further details on behavioral tests and imaging studies performed 1-year post-treatment and results, see Arias del Razo et al. (2022). All testing during and after treatment was uniform across all subjects included in the study.

### Pharmacological Treatments

We prepared treatments every month to ensure each subject received the appropriate dose based on their weight. For the OXT treatment, we dissolved oxytocin acetate salt (Santa Cruz Biotechnology, Dallas, Texas, USA) in saline at a concentration of 0.8 IU per kilogram of body weight (0.8 IU/kg = 1.6 μg/kg). Aliquots of the treatment solutions were stored in microcentrifuge tubes at −80°C until use.

We habituated and trained subjects using positive reinforcement techniques to all the steps required to receive an intranasal dose before the first day of the chronic treatment. Administration of chronic and acute treatments took less than 5 min per animal and took equal amounts of time for SAL and OXT groups. Doses were thawed before each use and kept cold, while trained personnel captured the subject from its home cage using a small transport box (60 cm × 30 cm × 30 cm), checked the animal’s identification number, and wrapped the subject using a towel. While being held, we administered 25 μl of OXT or SAL into each nostril using a manual single-channel pipette, alternating nostrils for a total of 50 μl. A thumb was placed individually over each nostril to prevent the expulsion of the compound and held for a few seconds until the compound was absorbed into the nasal mucosa. Pieces of banana were given to the monkey as a reward and the subject was returned to the home cage.

#### Chronic Treatment

For the chronic treatment, subjects received daily intranasal treatments (IN OXT or SAL) between 9:00 a.m. and 10:00 a.m. for 6 months. Treatment administration started at 12 months (360 ± 2 days) and continued until 18 months (540 ± 2 days) of age. The age interval encompasses the late pubertal period, as well as the late juvenile period (Valeggia et al., 1999). The age and dosage were based on clinical trials (ClinicalTrials.gov identifiers: NCT01908205 and NCT01308749) that were in progress at the time of the study.

#### Acute Treatment

Subjects received four acute treatments as adults (35 months of age), two IN OXT, and two SAL. Each dose of IN OXT (0.8 IU/KG) or SAL was administered at 10:30 a.m. on each testing day followed by an uptake period, a Brief Separation or a Non-Separation condition, and a Reunion period. Acute treatments were prepared and administered following the same procedures used during chronic treatment.

### Study Design

In order to assess the effects of a single dose of IN OXT or SAL in subjects with previous developmental exposure to IN OXT or SAL in the context of pair-mate involuntary brief separation, each subject went through four testing sessions, two control conditions or Non-Separation with each acute treatment: SAL/Non-Separation, OXT/Non-Separation, and two Brief Separations conditions: SAL/Brief Separation and OXT/Brief Separation (Supplementary Figure S1). Conditions and acute treatments were counterbalanced based on the chronic treatment group. We tested subjects at least 48 h apart and we only tested one animal per day to avoid bias in behavioral and physiological responses. Colony rooms were kept undisturbed for the duration of the test.

#### Brief Separation Condition

On the test day, the subject received a single dose of IN OXT or SAL at 10:30 a.m. and was returned to the home cage with its pair mate for a 30 min uptake period. After the uptake period, the pair mate was removed from the home cage using a transport box and taken to a different room. The subject stayed alone in the home cage inside the colony room. The subject was not in auditory or visual contact with its pair mate but was in auditory and visual contact with other colony animals. We video-recorded the subject’s locomotion and vocalizations during the Brief Separation period (30 min) as behavioral indices of agitation or distress. At the end of the Brief Separation period, we collected a blood sample from the subject to measure cortisol levels. The subject was caught from the home cage using a transport cage and manually restrained to obtain a blood sample (1 ml) *via* femoral puncture using 1-cc syringes pretreated with heparin. Blood samples were collected within 5 min after starting the capture procedure (cage entrance). The average time of blood collection was 2 min 46 s ± 8.8 s (mean ± standard error). Following blood collection, samples were immediately placed on ice, centrifuged at 1,610× *g* at 4°C, and the plasma extracted and stored at −80°C until assay. The subject and pair mate were returned to their home cage after the blood draw was completed and the pair was filmed for 15 min to be scored later for social behaviors (Supplementary Figure S2A; ethogram defined in Table 1).

**Table 1:** Reunion ethogram for social behaviors.

**Behavior**	**Definition**
Proximity	Subject’s body (excluding the tail) is within arm’s length (approximately 6 inches) of the partner (excluding the tail).
Contact	Subject and pair mate’s bodies are touching.
Tail twinning	Tails are intertwined at least one turn (score in addition to contact)
Grooming	The subject is grooming the pair mate (score in addition to contact)
Being groomed	The pair mate is grooming the subject (score in addition to contact)

#### Non-separation Condition

On the test day, the subject received a single dose of IN OXT or SAL at 10:30 a.m. and was returned to the home cage with its pair mate for a 30 min uptake period. After the uptake period, both the subject and the pair mate stayed together in the home cage for 30 min. The pair was in auditory and visual contact with other colony animals. We video-recorded the subject’s locomotion and vocalizations during the Non-Separation period (30 min). At the end of the Non-Separation period, we collected, processed, and stored the subject’s blood sample following the same procedure mentioned above for the Brief Separation condition. After the blood draw was completed, the subject was returned to its home cage to its pair mate. The pair was filmed for 15 min to be scored later for social behaviors (Supplementary Figure S2B; ethogram defined in Table 1).

### Acoustic Data Collection

During the Brief Separation and Non-Separation periods, we recorded the subject’s vocalizations using a video camera and a Marantz PMD 660 flash recorder with a Marantz Professional Audio Scope SG-5B directional condenser microphone. Recordings collected with the flash recorder were made with a sampling rate of 44.1 Hz and 16-bit resolution and saved as Waveform (.wav) audio files. Audio taken during the video recording of each test (mp4) was also converted to Waveform (.wav) audio files for acoustic analysis. We collected recordings noninvasively from outside the subject’s home cage with a microphone positioned within 3 meters from the calling subject.

### Acoustic Analysis

We visualized and analyzed audio files by creating spectrograms using Raven Pro 1.5 Sound Analysis Software (K. Lisa Yang Center for Conservation Bioacoustics, Cornell Lab of Ornithology 2014, Ithaca, NY). Spectrograms were generated with a 512-point (11.6 ms) Hann window (3 dB bandwidth = 124 Hz), amounting to a frequency resolution of 93.8 Hz and a temporal resolution of 5.33 ms. Two raters were trained to manually select vocalizations from the spectrograms, and a subset of recordings (10%) analyzed by both raters was used to determine their inter-rater reliability greater than 95%.

**Figure 1:**
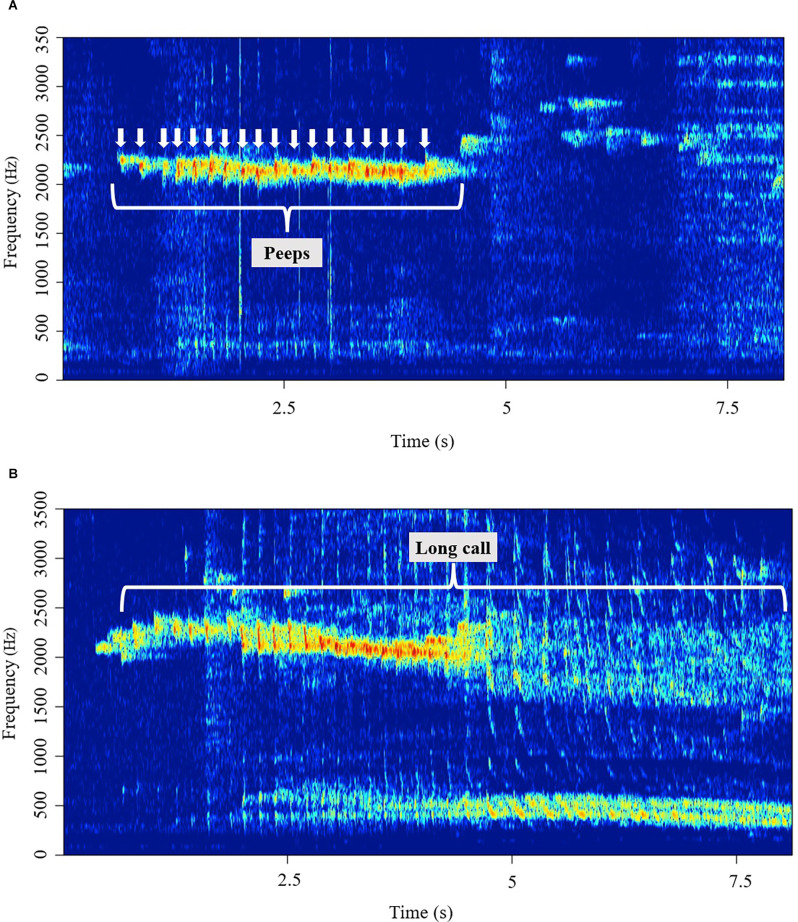
**(A)** A representative spectrogram of a series of peeps, repetitive pulse-like vocalizations. **(B)** A representative spectrogram of a long call, wideband vocalizations with a prominent low-frequency component.

We classified vocalizations into three categories: “peeps”, “long calls”, and “others”. Peeps are short (<100 ms), simple, repetitive pulse-like vocalizations that usually occur in a succession of multiple pulse notes centered around 5.5 kHz (Figure 1A). Long calls are complex, wideband vocalizations with a prominent low-frequency component, as seen in Figure 1B. Peeps were individually counted, while long calls were scored by their length (duration). Vocalizations that did not fall in either of the two categories above were classified as “other” and were not considered in this study.

Since vocalizations were recorded in a room that houses multiple titi monkey families, spectrograms of the subject were analyzed side-by-side with the corresponding videos of the Brief Separation or Non-Separation conditions to confirm that vocalizations came from the subject. Since titis have sometimes been observed to produce vocalizations—particularly peeps—with little bodily movement, a combination of the spectrogram and at least two other factors was used to identify when the subject of interest was vocalizing. Lip movements were the most direct visual cue of vocalization, followed by rhythmic movements of the torso that temporally coincided with auditory cues. Temporal and tonal qualities of vocalizations previously attributed to an individual with high confidence (e.g., when an individual’s vocalizations were observed to consistently fall within a specific frequency band, or when a temporally unique feature not observed in other individuals was present) were used to assign a vocalization in the absence of visual cues, or when multiple monkeys vocalized simultaneously. In the latter case, perceived interaural intensity differences were also considered when identifying the source of vocalization (Agamaite et al., 2015).

### Behavioral Data Collection

During the Brief Separation and Non-Separation periods, subjects’ behavior was video recorded from outside the subject’s home cage. Locomotion was later scored by two validated scorers (>95% inter-rater reliability) using Behavior Tracker 1.5^1^. The subject’s locomotion duration was scored every time the subject moved at least one body length and continued until the subject was in an immobile position for at least 2 s.

During the Reunion period, the subject and pair mate’s behavior was video recorded. The video was later scored by two validated observers (>95% inter-rater reliability) for social behavior according to the ethogram in Table 1 using Behavior Tracker 1.5^1^.

### Hormone Assessment

Blood samples were centrifuged at 4°C for 15 min. Plasma was extracted from the centrifuged sample and stored at −80°C until assay. Plasma cortisol was assayed at the UC Davis Endocrinology Laboratory, using an enzyme immunoassay previously validated both chemically and biologically for titi monkeys, and described in detail elsewhere (Witczak et al., 2021). Inter-assay CVs were 3.9% and intra-assay CVs were 3.0%.

### Data Analysis

For the vocalization analysis, we only considered the data for the Brief Separation condition. Condition (Brief Separation and Non-Separation) was not considered as an independent variable for vocalizations (peeps and long calls) because of the lack of vocalizations in the Non-Separation condition data. For the Non-Separation condition, the average number of peeps was 2 (range: 0–51). No long calls occurred during Non-Separation. For the rest, of the data sets (locomotion, cortisol, behavioral data), condition (Brief Separation and Non-Separation) was considered as an independent variable.

We tested for normality and equal variances using the Shapiro-Wilk test and Bartlett test of homogeneity of variances. Peeps (number) and long calls (duration) did not fulfill these assumptions. We performed a GLMM with a Poisson distribution using the *lmer* function from the *lme4* R package (Pinheiro and Bates, 2021) for each variable. IN treatment (OXT, SAL), and sex (females, males) were independent variables (fixed), and the subject was a random factor. All p-values below 0.05 were considered significant. Bonferroni corrections for multiple comparisons were carried out for pairwise comparisons between means. Figures show means with standard error bars.

For locomotion, we transformed the data to ranks and performed a *nlme*: Linear and Nonlinear Mixed Effects Models package (Pinheiro and Bates, 2021) in R. IN treatment (OXT, SAL), condition (Brief Separation and Non-Separation), and sex (females, males) were independent variables (fixed), and the subject was a random factor. All p-values below 0.05 were considered significant. Bonferroni corrections for multiple comparisons were carried out for pairwise comparisons between means.

For cortisol, we only considered males and non-pregnant females, as cortisol levels can be affected during gestation (6 females were pregnant during the study). We performed a *nlme*: Linear and Nonlinear Mixed Effects Models package (Pinheiro and Bates, 2021) in R. IN treatment (OXT, SAL), condition (Brief Separation and Non-Separation), and sex (females, males) were independent variables (fixed), and the subject was a random factor. All p-values below 0.05 were considered significant. Bonferroni corrections for multiple comparisons were carried out for pairwise comparisons between means.

For contact and proximity, we performed *nlme*: Linear and Nonlinear Mixed Effects Models package (Pinheiro and Bates, 2021) in R. IN treatment (OXT, SAL), condition (Brief Separation and Non-Separation) and sex (females, males) were independent variables (fixed), and the subject was a random factor. All p-values below 0.05 were considered significant. Bonferroni corrections for multiple comparisons were carried out for pairwise comparisons between means. Figures show means with standard error bars.

The variables tail twining, grooming, and being groomed were consolidated into one variable named active contact, to reduce the number of zeros present in the data. The variable active contact did not fulfill assumptions of normality or homogeneity of variances. We performed a GLMM with a Poisson distribution. IN treatment (OXT, SAL), condition (Brief Separation and Non-Separation), and sex (females, males) were independent variables (fixed), and the subject was a random factor. All p-values below 0.05 were considered significant. Bonferroni corrections for multiple comparisons were carried out for pairwise comparisons between means. Figures show means with standard error bars.

## Results

### Vocalizations: Peeps (Number)

**Figure 2:**
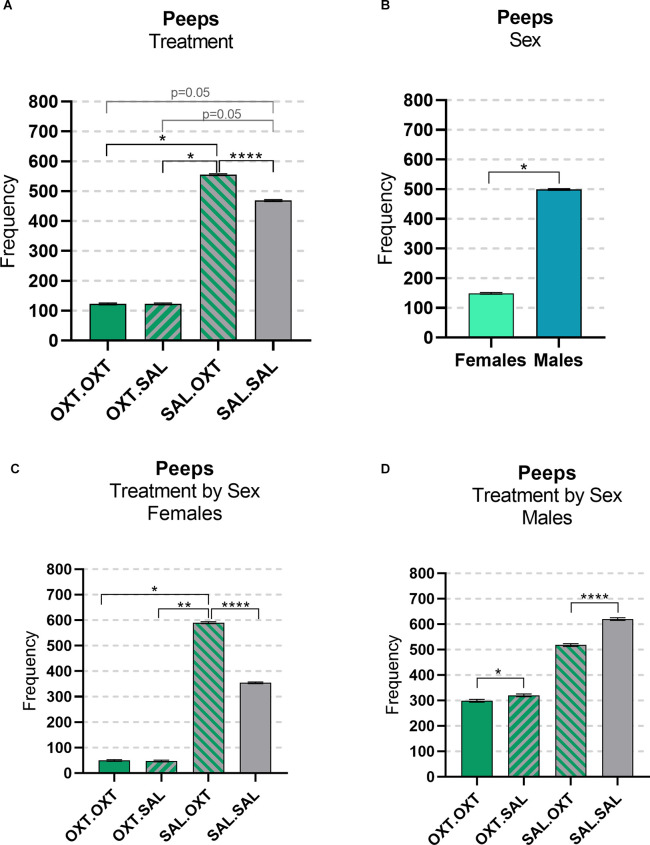
Vocalizations: peeps. **(A)** OXT.OXT and OXT.SAL treated subjects vocalized less peeps than SAL.OXT subjects, SAL OXT subjects vocalized the most compared to other groups. **(B)** Females treated with OXT.OXT and OXT.SAL females vocalized less peeps than SAL. OXT females, SAL OXT females vocalized the most compared to all female groups. **(C)** Females treated with OXT.OXT or OXT.SAL had fewer peeps than females treated with SAL.OXT. Overall, SAL.OXT females vocalized the most peeps. **(D)** OXT.OXT males vocalized fewer peeps than OXT.SAL males. SAL.OXT males vocalized fewer peeps than SAL.SAL males. Significance summary: *p < 0.05, **p ≤ 0.01, ****p ≤ 0.0001.

We found significant main effects for treatment (*F*_3,47_ = 23.782, *p* < 0.00001), sex (*F*_1,47_ = 4.172, *p* = 0.0122), and treatment by sex interaction (*F*_3,47_ = 153.35, *p* < 0.0001). Subjects treated with chronic OXT (OXT.OXT and OXT.SAL) vocalized significantly fewer peeps than subjects that received chronic SAL and acute OXT (SAL.OXT) and showed a trend for vocalizing fewer peeps than SAL.SAL. Overall, SAL.OXT subjects vocalized the most peeps during the Brief Separation (Figure 2A; see Table 2 for pairwise comparations). Males vocalized more peeps than females during the Brief Separation condition (*β* = 2.8, *p* = 0.049; Figure 2B).

**Table 2:** Vocalizations. Peeps (number) and long call (duration) comparations by treatment.

**Treatment**	**β**	**SE**	**P value**
**Peeps**			
OXT.OXT—OXT.SAL	1.01	0.02	0.985
OXT.OXT—SAL.OXT	4.52	0.52	0.02^*^
OXT.OXT—SAL.SAL	3.82	0.52	0.051
OXT.SAL—SAL.OXT	4.48	0.524	0.021^*^
OXT.SAL—SAL.SAL	0.26	0.524	0.054
SAL.OXT—SAL.SAL	1.18	0.016	<0.0001^****^
**Long calls**			
OXT.OXT—OXT.SAL	0.362	0.4163	0.0605
OXT.OXT—SAL.OXT	0.621	0.0764	<0.0001^****^
OXT.OXT—SAL.SAL	5.930	0.5252	<0.0001^****^
OXT.SAL—SAL.OXT	0.220	0.4172	0.0016^***^
OXT.SAL—SAL.SAL	3.688	0.5260	<0.0001^****^
SAL.OXT—SAL.SAL	0.0268	0.3272	0.4460

Significance summary: ^*^p < 0.05, ^***^p ≤ 0.001, ^****^p ≤ 0.0001.

Females treated with chronic OXT and acute OXT (OXT.OXT) or SAL (OXT.SAL) produced fewer peeps than females treated with chronic SAL and acute OXT (SAL.OXT). Overall, SAL.OXT females vocalized the most peeps during the Brief Separation (Figure 2C; see Table 3 for pairwise comparations).

**Table 3:** Vocalizations: peeps and long calls pairwise comparations by sex and treatment.

**Treatment**	**β**	**SE**	**P value**
**Females peeps**			
Females OXT.OXT—Females OXT.SAL	0.047	0.045	0.965
Females OXT.OXT—Females SAL.OXT	0.840	0.745	0.02^*^
Females OXT.OXT—Females SAL.SAL	0.14	0.745	0.145
Females OXT.SAL—Females SAL.OXT	2.225	0.745	0.01^**^
Females OXT.SAL—Females SAL.SAL	0.134	0.745	0.124
Females SAL.OXT—Females SAL.SAL	1.67	1.025	<0.0001^****^
**Males peeps**			
Males OXT.OXT—Males OXT.SAL	0.937	1.021	0.04*
Males OXT.OXT—Males SAL.OXT	0.581	0.737	0.995
Males OXT.OXT—Males SAL.SAL	−0.721	0.737	0.977
Males OXT.SAL—Males SAL.OXT	−0.477	0.737	0.998
Males OXT.SAL—Males SAL.SAL	−0.657	0.737	0.987
Males SAL.OXT—Males SAL.SAL	0.836	1.021	<0.0001^****^
**Female long calls**			
Females OXT.OXT—Females OXT.SAL	0.0197	0.140	0.903
Females OXT.OXT—Females SAL.OXT	0.0294	0.628	0.516
Females OXT.OXT—Females SAL.SAL	0.721	0.897	0.054
Females OXT.SAL—Females SAL.OXT	0.246	0.629	0.333
Females OXT.SAL—Females SAL.SAL	0.131	0.898	0.08
Females SAL.OXT—Females SAL.SAL	0.067	0.574	0.545
**Males long calls**			
Males OXT.OXT—Males OXT.SAL	0.125	0.062	0.5733
Males OXT.OXT—Males SAL.OXT	0.003	0.546	0.7733
Males OXT.OXT—Males SAL.SAL	0.097	0.546	0.3606
Males OXT.SAL—Males SAL.OXT	0.410	0.547	0.008**
Males OXT.SAL—Males SAL.SAL	0.1349	0.547	0.06
Males SAL.OXT—Males SAL.SAL	0.712	0.045	<0.0001^****^

Significance summary: ^*^p < 0.05, ^**^p ≤ 0.01, ^****^p ≤ 0.0001.

Males treated with chronic OXT and acute OXT (OXT.OXT males) vocalized fewer peeps than when they received acute SAL (OXT.SAL males). SAL.OXT males vocalized fewer peeps than SAL.SAL males (Figure 2D; see Table 3 for pairwise comparations).

### Vocalizations: Long Calls (Duration)

**Figure 3:**
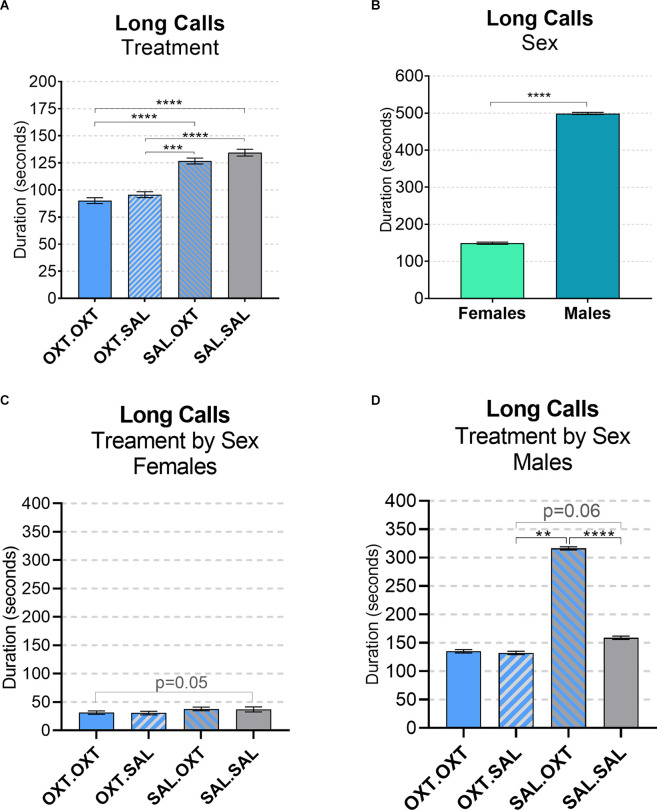
Vocalizations: long calls. **(A)** Subjects from chronic OXT treatment groups (OXT.OXT and OXT.SAL) had a shorter duration of long calls than subjects from chronic SAL treatment groups (SAL.OXT and SAL.SAL). **(B)** Males had longer long call durations than females. **(C)** Non-significant trend for OXT. OXT females have shorter long calls than SAL.SAL females. **(D)** Males treated with SAL. OXT had a longer duration of long calls than males treated with OXT.SAL and SAL.SAL. Significance summary: **p ≤ 0.01, ***p ≤ 0.001, ****p ≤ 0.0001.

We found significant main effects for treatment (*F*_3,52_ = 68.13, *p* < 0.002), sex (*F*_1,51_ = 43.36, *p* < 0.01), and treatment by sex interaction (*F*_3,48_ = 19.53, *p* < 0.01). Subjects from chronic OXT treatment groups (OXT.OXT and OXT.SAL) had shorter durations of long calls than subjects from chronic SAL treatment groups (SAL.OXT and SAL.SAL; Table 2; Figure 3A). The average duration of long calls was significantly shorter for females compared to males (*β* = 0.561, *p* < 0.0001; Figure 3B). We found a non-significant trend for OXT.OXT females having shorter long calls than SAL.SAL females (Table 3; Figure 3C). Males treated with SAL.OXT had the longest duration of long calls compared to OXT.SAL and SAL.SAL (Table 3; Figure 3D).

### Locomotion

We found a significant main effect for locomotion by condition (*F*_1,59_ = 11.94, *p* = 0.001), with subjects moving more during the Brief Separation (232.05 ± 17.42) than in the Non-Separation condition (159.9 ± 21.44; *t* = −3.440, *p* = 0.0011). There were no significant effects by treatment (*F*_3,59_ = 0.586, *p* = 0.626) or sex (*F*_1,59_ = 0.09, *p* = 0.756), or treatment by sex interaction (*F*_1,59_ = 0.607, *p* = 0.613; see Supplementary Table S1 and Supplementary Table S1.1 for mean and SEs).

### Cortisol

We found a significant main effect for condition, with cortisol levels being lower during the Non-Separation condition (321 ± 77.82 ng/ml) compared to the Brief Separation condition (446 ± 74.1 ng/ml; *F*_1,42_ = 6.947, *p* < 0.01). There were no significant effects by treatment (*F*_3,42_ = 0.726, *p* = 0.54) or sex (*F*_1,21_ = 0.411, *p* = 0.528) or treatment by sex by condition interaction (*F*_3,42_ = 0.45, *p* = 0.713; see Supplementary Table S2 and Supplementary Table S2.1 for means and SEs).

### Reunion Behavior

#### Contact

**Figure 4:**
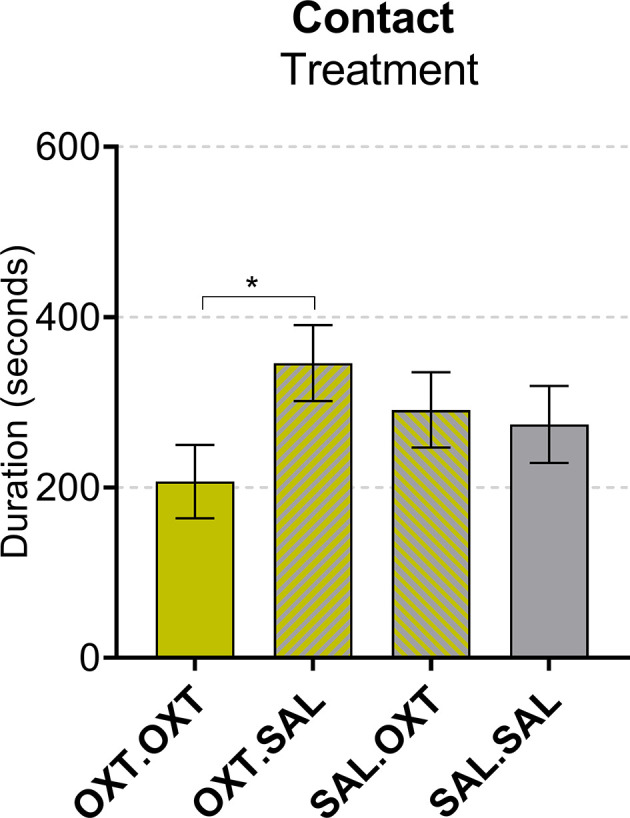
Contact during reunion. Subjects treated with chronic OXT and acute OXT spent less time in contact with their pair mate following reunion compared to subjects that received chronic OXT and acute SAL. Significance summary: *p < 0.05.

We found a main effect by treatment (*F*_3,55_ = 3.67, *p* = 0.017). Subjects that received chronic OXT plus acute OXT spent less time in contact with their pair mate following Reunion compared to subjects that received chronic OXT and acute SAL (*β* = −139.1, *p* = 0.028; Figure 4).

#### Proximity

We found no significant results for treatment (*F*_3,26_ = 0.814, *p* = 0.355), sex (*F*_1,26_ = 0.381, *p* = 0.54) or treatment by sex interaction (*F*_3,26_ = 0.702, *p* = 0.555).

#### Active Contact

**Figure 5:**
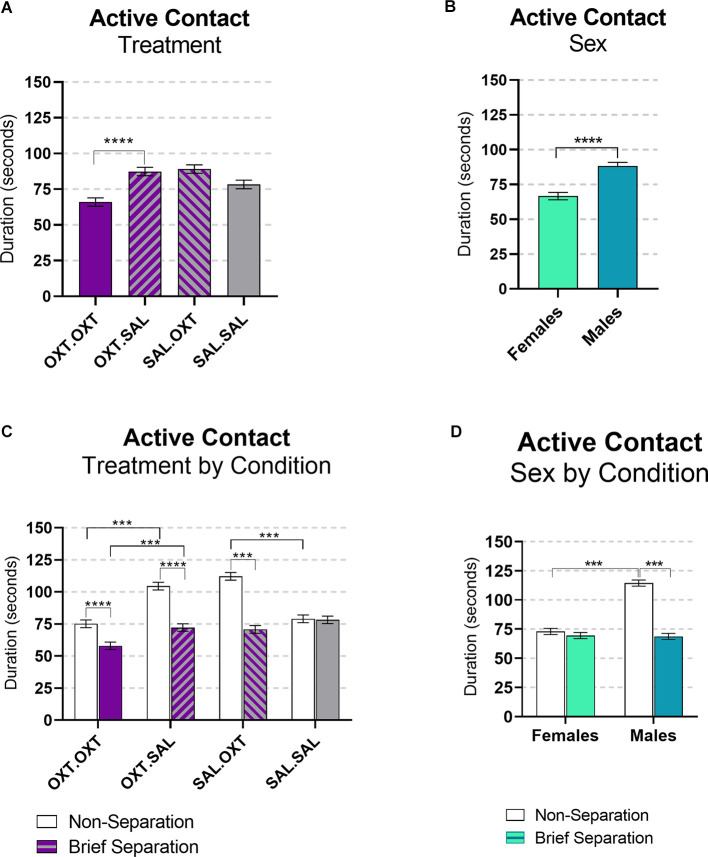
Active contact during reunion. **(A)** Subjects treated OXT.OXT displayed less active contact behaviors compared to subjects that received OXT.SAL. **(B)** Males displayed more active contact than females. **(C)** All the treatment groups displayed significantly less active contact during the brief Separation condition compared to the Non-Separation, except SAL.SAL. Following a Non-Separation, acute OXT in animals treated with chronic SAL increased active contact compared to acute SAL. In animals treated with chronic OXT, acute OXT decreased active contact compared to acute SAL. **(D)** Males displayed more active contact during the Non-Separation Condition compared to the Separation condition and compared to females in the Non-Separation Condition. Significance summary: ***p ≤ 0.001, ****p ≤ 0.0001.

This variable includes tail twining, grooming, and being groomed. We found significant main effects by treatment and sex, and interactions for sex by condition and treatment by condition. Subjects treated with chronic OXT and acute OXT (OXT.OXT) displayed less active contact behaviors compared to subjects that received chronic OXT and acute SAL (OXT.SAL; *β* = −0.75, *p* < 0.0001; Figure 5A). Overall, male subjects displayed more active contact than female subjects (*β* = 1.24, *p* < 0.0001; Figure 5B). All the treatment groups significantly displayed less active contact following the Separation compared to the Non-Separation condition, except SAL.SAL (OXT.OXT: *β* = 1.294, *p* < 0.0001; OXT.SAL: *β* = 1.46, *p* < 0.0001; SAL.OXT: *β* = 1.583, *p* < 0.0001; SAL.SAL: *β* = 0.013, *p* = 1.00). Following a Non-Separation condition, acute OXT in animals treated with chronic SAL increased active contact compared to acute SAL (*β* = 1.41, *p* < 0.0001). However, in animals treated with chronic OXT, acute OXT decreased active contact compared to acute SAL (*β* = 0.71, *p* < 0.0001; Figure 5C). Males displayed more active contact during Reunion that followed the Non-Separation condition compared to the Reunion following the Brief Separation condition (*β* = 1.66, *p* < 0.0001), and compared to females following the Non-Separation condition (*β* = −1.568, *p* < 0.0001; Figure 5D).

## Discussion

The goal of our study was to investigate the chronic and acute effects of IN OXT on separation distress and affiliation upon reunion in adult pair-bonded titi monkeys. Our subjects were previously treated with chronic IN OXT or SAL during the peri-adolescent period. Here we examined the subjects’ response to a brief separation from their pair mate 30 min after receiving a single dose of IN OXT or SAL and their behavior upon reunion. We found that both acute and chronic IN OXT treatments had effects on vocalizations during the Brief Separation period and on contact during the Reunion phase. Chronic OXT subjects overall had fewer vocalizations during the Brief Separations and lower levels of contact with their pair mates upon Reunion, suggesting that chronic OXT treatment blunts the separation response. Subjects treated with chronic SAL and acute OXT had sexually dimorphic effects; for females, it appeared to increase the vocalization response to separation (increased frequency of peeps), while in males the vocalization response reflected an increase in partner seeking behavior (increased duration of long calls). Cortisol and locomotion were significantly higher during the Separation period compared to the Non-Separation period. However, contrary to what we expected, we did not find treatment or sex effects in cortisol or locomotion. It is possible that locomotion, cortisol, and vocalizations are different parts of the stress response and are affected differentially by IN OXT (i.e., temporally).

Other studies using IN OXT in non-human primates have also found no significant effects on cortisol. Parker et al. (2005) examined the stress response to acute social isolation (30 and 90 min) in squirrel monkeys, IN OXT attenuated the ACTH response to stress (at 90 min) but did not affect cortisol levels (at 30 min or 90 min). This result suggests that IN OXT might not directly attenuate the adrenal stress response, or that the assessment period was too short to capture the treatment (which could also be the case in our study) as the adrenal response to stress temporally follows that of the pituitary. Cavanaugh et al. (2016) found that in white-tufted ear marmosets IN administration of OXT did not alter cortisol or behavior in response to a stressor; however, marmosets treated with an oral OXT antagonist had higher HPA-axis activity, resulting in altered cortisol levels and behavior. Both male and female marmosets treated with an OXT antagonist spent significantly less time in close proximity to their mate during the first 30 min of the stressor than when they were treated with saline, suggesting that the OXT system may be important for the expression of partner-seeking behavior during a stressor.

### Effects of Chronic IN OXT Administration

Chronic IN OXT led to a dampened response to separation in both females and males, reflected in lower frequencies of peep calls and shorter durations of long calls while separated, and less time in contact during the Reunion. This response was more accentuated in females, producing the lowest frequency of peeps. In our previous study (Arias del Razo et al., 2022) examining the long-term effects of chronic OXT on adult pair-bonding behavior, we found that chronic IN OXT females had a temporary delay in forming a partner preference at 1 week post-pairing compared to chronic IN SAL subjects and chronic IN OXT males. However, at 4 months post-pairing all groups had successfully formed a preference for their partner. This initial delay in partner preference formation, the reduced vocalizations during the Brief Separations, and low levels of contact upon reunion suggest that females treated with chronic OXT have a lower quality pair bond, with chronic IN OXT affecting behaviors during the formation and maintenance of the bond. These results are in contrast to findings reported in female prairie voles where the same dose of chronic IN OXT (0.8 IU/kg) during development appeared to facilitate pair bonding in adult females (Bales et al., 2013).

### Effects of Acute OXT Administration

The effects of acute OXT administration were somewhat different in males and females. In males that had received chronic SAL, acute OXT decreased the number of peeps while increasing the duration of long calls. The functions of peeps and long calls are somewhat different. Peeps are shorter-range calls that mostly occur when an individual is isolated or in distress, their function in the wild is for locating group members and aiding the cohesiveness of the pair/group and are often accompanied by arousal or anxiety-like behaviors (Robinson, 1979). Long calls represent the duet contributions that are sung by the male and female each morning (Robinson, 1979), and due to a lower frequency, likely reach a farther distance (Robinson, 1981). Long calls contain information on the individual identity of the caller, reinforce the pair bond, and are used for maintaining territories, intragroup cohesion, and mate attraction (Robinson, 1981; Snowdon, 1988; Lau et al., 2020). It is possible that acute OXT-treated males experienced lower anxiety upon separation, resulting in fewer peeps, and increased partner seeking behavior reflected in longer duration long calls (a long-range strategy to regain contact).

In females that had received chronic SAL, acute OXT led to an increase in peeps with no change in the duration of long calls. These results suggest that in females, acute exogenous OXT is potentiating the separation response. This is contrary to our hypothesis, as acute OXT was expected to produce an anxiolytic response. However, it is not uncommon for IN OXT to have sex-specific effects in animal models and humans (Ditzen et al., 2013; Steinman et al., 2016), particularly on stress, anxiety, and negative emotional stimuli (Love, 2018; Hale et al., 2021). Additionally, other studies have reported anxiogenic action of acute OXT in other contexts (Grillon et al., 2013; Jang et al., 2021).

### Reunion Period

Subjects treated with chronic IN OXT and acute OXT displayed lower levels of contact and active contact (following a Brief Separation and a Non-Separation) with their pair mates compared to acute SAL upon reunion. These detrimental effects of chronic IN OXT in social behavior can be potentially explained by prolonged over-stimulation of the OXT system, causing OXT receptors (OXTRs) to undergo desensitization and internalization (Gimpl and Fahrenholz, 2001; Conti et al., 2009). Chronic IN OXT treatment in mice has led to decreased OXTRs throughout the areas involved in the regulation of social recognition [LS, HIPP, NACC, AMY, piriform cortex (PC), and anterior olfactory nucleus AON] while having less impact on V1aR (Huang et al., 2014).

Following a Non-Separation condition and the blood draw, acute OXT in animals treated with chronic SAL increased active contact during the Reunion Period, compared to acute SAL. This result paired with the increased duration of long calls displayed by subjects treated with chronic SAL and acute OXT, particularly males, provides more evidence that acute OXT in animals with no previous OXT exposure increased partner-seeking behavior. These results align with the findings from Cavanaugh et al. (2018) that showed marmosets treated with an oxytocin antagonist spent less time in proximity with their pair mate upon reunion following a long-separation challenge. These findings contribute to the literature indicating that the OXT system is critical to behavioral processes that contribute to the preservation of long-lasting social bonds.

## Conclusions

Both acute and chronic OXT had effects on the separation response, primarily on vocalizations during the Brief Separation and on contact during the Reunion phase. While chronic OXT treatment administered during development dampened the separation response, chronic SAL and acute OXT had sexually dimorphic effects in which they appeared to increase the response to separation in females, while increasing pair mate-seeking behavior in males. Cortisol and locomotion were significant by condition, but we did not find any treatment or sex effects. The impact of chronic and acute manipulations of IN OXT on the response to an involuntary brief separation and the expression of affiliation upon reunion depended on the previous exposure to either OXT or SAL, sex, and context.

## Data Availability Statement

The data supporting the conclusions of this study are available from the corresponding author upon reasonable request.

## Ethics Statement

The animal study was reviewed and approved by UC Davis’ Institutional Animal Care and Use Committee (IACUC).

## Author Contributions

KB conceived and designed the overall intranasal oxytocin study, and wrote sections of the first draft. RA designed the study described in this manuscript, collected data, analyzed data, and wrote most of the first draft and final draft. MV statistically analyzed the data. ML, PT, TW, and LG contributed to data collection, behavioral scoring, and project administration. PT and AL analyzed vocalization data. All authors contributed to the article and approved the submitted version.

## Conflict of Interest

The authors declare that the research was conducted in the absence of any commercial or financial relationships that could be construed as a potential conflict of interest.

## Publisher’s Note

All claims expressed in this article are solely those of the authors and do not necessarily represent those of their affiliated organizations, or those of the publisher, the editors and the reviewers. Any product that may be evaluated in this article, or claim that may be made by its manufacturer, is not guaranteed or endorsed by the publisher.

## Footnotes

^1^ www.behaviortracker.com
