# Supplementary material for: Effects of Chronic and Acute Intranasal Oxytocin Treatments on Temporary Social Separation in Adult Titi Monkeys (Plecturocebus cupreus)
Source: Front Behav Neurosci. 2022 Jun 22;16:877631. doi: 10.3389/fnbeh.2022.877631 (PMC9257099; doi:10.3389/fnbeh.2022.877631)
Supplement: Supplementary file 1 [file Data_Sheet_1.docx]

Supplementary Material

**Figure S1**. Study design. Subjects that received chronic treatment (OXT or SAL) during development (12 to 18 months of age) and four acute treatments as adults (35 months of age) followed by Separation or Non-Separation conditions.


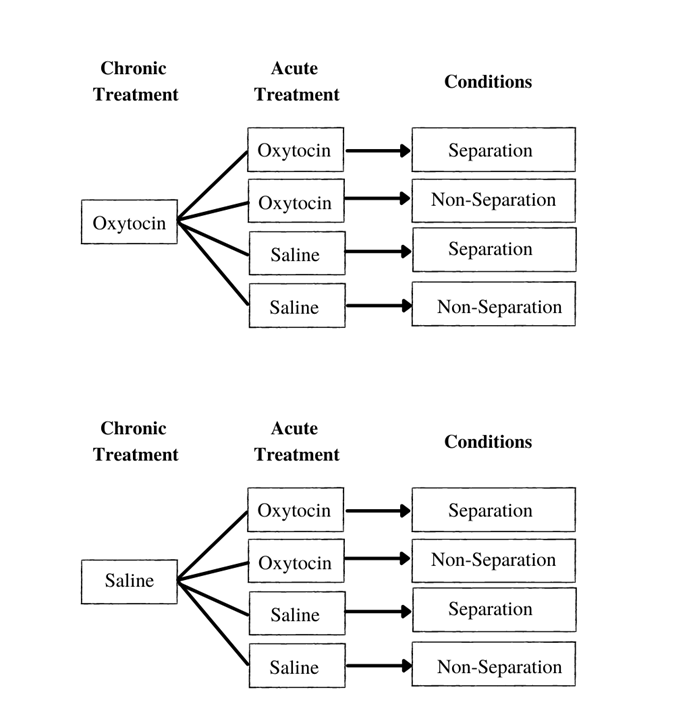


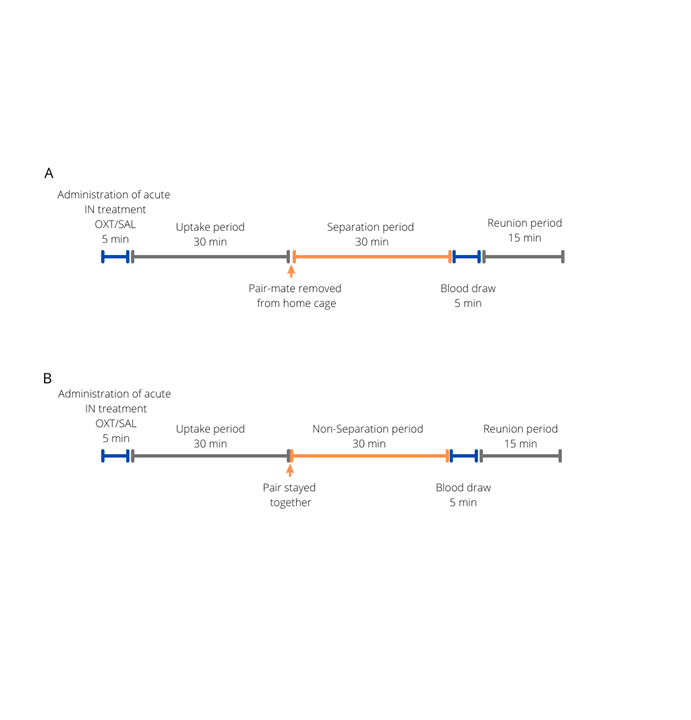
**Figure S2** **A.** Separation condition timeline B. Non-Separation condition timeline.

**Table S1. Locomotion.** Means and standard errors for treatment by condition interaction.

| **Condition** | **Treatment** | **Means** | **SE** |
| --- | --- | --- | --- |
| **No Separation** | OXT.OXT | 191 | 45 |
|  | OXT.SAL | 162 | 43.1 |
|  | SAL.OXT | 169 | 41.1 |
|  | SAL.SAL | 127 | 45.9 |
| **Separation** | OXT.OXT | 247 | 36.9 |
|  | OXT.SAL | 216 | 36.9 |
|  | SAL.OXT | 244 | 39.6 |
|  | SAL.SAL | 221 | 39.6 |

**Table S1.1 Locomotion.** Means and standard errors for treatment, by condition, by sex interaction.

| **Condition** | **Sex** | **Treatment** | **Means** | **SE** |
| --- | --- | --- | --- | --- |
| **No Separation** | **Females** | OXT.OXT | 163.4 | 63.6 |
|  |  | OXT.SAL | 181.1 | 58.1 |
|  |  | SAL.OXT | 197.7 | 58.1 |
|  |  | SAL.SAL | 164.1 | 71.1 |
|  | **Males** | OXT.OXT | 218 | 63.6 |
|  |  | OXT.SAL | 142.6 | 63.6 |
|  |  | SAL.OXT | 140.7 | 58.1 |
|  |  | SAL.SAL | 89.5 | 58.1 |
| **Separation** | **Females** | OXT.OXT | 228.7 | 50.4 |
|  |  | OXT.SAL | 209.4 | 50.4 |
|  |  | SAL.OXT | 270.3 | 58.2 |
|  |  | SAL.SAL | 164.3 | 58.2 |
|  | **Males** | OXT.OXT | 264.7 | 53.9 |
|  |  | OXT.SAL | 222.6 | 53.9 |
|  |  | SAL.OXT | 217.3 | 53.9 |
|  |  | SAL.SAL | 278.7 | 53.9 |

**Table S2. Cortisol.** Means and standard errors (ng/ml) for treatment by condition interaction.

| **Condition** | **Treatment** | **Means** | **SE** |
| --- | --- | --- | --- |
| **No Separation** | OXT.OXT | 367 | 74.2 |
|  | OXT.SAL | 303 | 74.4 |
|  | SAL.OXT | 300 | 79.4 |
|  | SAL.SAL | 317 | 83.3 |
| **Separation** | OXT.OXT | 427 | 71.4 |
|  | OXT.SAL | 592 | 69.3 |
|  | SAL.OXT | 365 | 76.6 |
|  | SAL.SAL | 401 | 79.4 |

**Table S2.2 Cortisol.** Means and standard errors (ng/ml) for treatment, by condition, by sex interaction.

| **Condition** | **Sex** | **Treatment** | **Means** | **SE** |
| --- | --- | --- | --- | --- |
| **No Separation** | **Females** | OXT.OXT | 392 | 105.2 |
|  |  | OXT.SAL | 275 | 105.7 |
|  |  | SAL.OXT | 298 | 118.7 |
|  |  | SAL.SAL | 319 | 118.7 |
|  | **Males** | OXT.OXT | 342 | 104.8 |
|  |  | OXT.SAL | 331 | 104.8 |
|  |  | SAL.OXT | 302 | 105.4 |
|  |  | SAL.SAL | 315 | 116.8 |
| **Separation** | **Females** | OXT.OXT | 455 | 105.7 |
|  |  | OXT.SAL | 695 | 105.7 |
|  |  | SAL.OXT | 367 | 118.7 |
|  |  | SAL.SAL | 424 | 118.7 |
|  | **Males** | OXT.OXT | 398 | 96.1 |
|  |  | OXT.SAL | 488 | 89.7 |
|  |  | SAL.OXT | 363 | 96.9 |
|  |  | SAL.SAL | 378 | 105.4 |
